# Invasion by Cordgrass Increases Microbial Diversity and Alters Community Composition in a Mangrove Nature Reserve

**DOI:** 10.3389/fmicb.2017.02503

**Published:** 2017-12-15

**Authors:** Min Liu, Zheng Yu, Xiaoqing Yu, Yuanyuan Xue, Bangqin Huang, Jun Yang

**Affiliations:** ^1^Aquatic EcoHealth Group, Key Laboratory of Urban Environment and Health, Institute of Urban Environment, Chinese Academy of Sciences, Xiamen, China; ^2^University of Chinese Academy of Sciences, Beijing, China; ^3^College of Environment and Ecology, Xiamen University, Xiamen, China; ^4^Department of Chemical Engineering, University of Washington, Seattle, WA, United States

**Keywords:** *Spartina alterniflora*, biological invasion, bacterial diversity, community assembly, conditionally rare taxa, neutral process

## Abstract

Invasion by exotic plant species can alter ecosystem function and reduce native plant diversity, but relatively little is known about their effects on belowground microbial communities. Here we investigated the effects of exotic cordgrass (*Spartina alterniflora*) invasion on the distribution of soil bacterial communities in a mangrove nature reserve of the Jiulong River Estuary, southeast China using high-throughput sequencing of 16S rRNA gene and multivariate statistical analysis. Our results showed that *S. alterniflora* invasion altered soil properties, and significantly increased soil bacterial taxa richness, primarily by stimulating an increase in conditionally rare or rare taxa, and changes in community composition and function. Abundant, conditionally rare and rare subcommunities exhibited similar response patterns to environment changes, with both conditionally rare and rare taxa showing a stronger response than abundant ones. Habitat generalists were detected among abundant, conditionally rare and rare taxa, whereas habitat specialists were only identified among conditionally rare taxa and rare taxa. In addition, we found that vegetation was the key factor driving these patterns. However, our comparative analysis indicated that both environmental selection, and neutral process, significantly contributed to soil bacterial community assembly. These results could improve the understanding of the microbial processes and mechanisms of cordgrass invasion, and offer empirical data of use in the restoration and management of the mangrove wetlands.

## Introduction

The human-mediated introduction of invasive plants has altered both the biodiversity and stability of ecosystems worldwide. These invasive plants can be increasingly expensive to control, particularly under pressures of global environmental change (Jackson et al., 2001; Wolfe and Klironomos, 2005; Bu et al., 2015; Hobbie, 2015). *Spartina alterniflora*, which is highly invasive species of cordgrass, as demonstrated by its successful introductions around the world, was first introduced into China in 1979 (An et al., 2007; Yu et al., 2014b). Its distribution in Fujian province is more extensive than in other Chinese coastal provinces, and its expansion is replacing mangroves in the estuary of the Jiulong River at an alarming rate over recent years. This estuary is considered an important but fragile ecosystem, providing valuable ecosystem services to humans and other organisms (Wu et al., 2002; Wan et al., 2009; Yu et al., 2014b). Therefore there is an urgent need to understand the impacts of this invader on coastal ecosystem structure and function in this important area.

Although soil bacterial communities play important roles in ecosystem-level processes, most works on the effects of *S. alterniflora* invasions have focused on the plants properties, aboveground flora and fauna, abundant taxa and special soil microbial taxa associated with carbon, nitrogen and sulfur cycles (Wolfe and Klironomos, 2005; Liao et al., 2007; Zhou et al., 2009; Zhang et al., 2013b; Lin et al., 2015). Unfortunately, little is known about how *S. alterniflora* affects the rare bacterial subcommunities in the soils because it has been hard to study (e.g., by denaturing gradient gel electrophoresis (DGGE), clone library), the rare biosphere albeit its significant contribution to the cycling of particular elements such as nitrogen or sulfur (Pedrós-Alió, 2012; Hong et al., 2015). Currently, the development of high-throughput and deeper sequencing is allowing a much more direct identification, and increasing interest in, the rare biosphere community (Lekberg et al., 2013; Pholchan et al., 2013; Shade et al., 2014; Aanderud et al., 2015; Liu et al., 2015; Lynch and Neufeld, 2015). Recent studies indicated that abundant and rare taxa have similar spatial patterns, but do not contribute equally to the community variation because of differences in their ecological niches, role and intrinsic properties (Campbell et al., 2011; Liu et al., 2015; Chen et al., 2017; Dai et al., 2017). Other studies have provided detailed evidence of dynamic variation for rare taxa, implying that some rare taxa may be inactive or even permanently dormant, while others may conditionally bloom under favorable environmental conditions and conduct important ecological processes (Pedrós-Alió, 2012; Shade et al., 2014). However, the way rare bacterial taxa change after *S. alterniflora* invasion is currently unclear. In this study, we hypothesized that rare taxa do not respond in the same way as the abundant taxa to *S. alterniflora* invasion. The study of rare biosphere variation may give a better understanding of the influence of *S. alterniflora* invasion on soil bacterial community and ecosystem function.

Generally, bacterial communities are simultaneously influenced by both niche-based (e.g., environmental selection and niche partitioning) and neutral-based (e.g., ecological drift) processes. However, the relative importance of these processes in community variation remains difficult to resolve (Hanson et al., 2012; Logares et al., 2013). Several factors such as disturbance, habitat connectivity and size, predation, and resource availability have diverse and complex influences on the relative importance of niche-based vs. neutral-based processes in the assembly of bacterial communities (Zhou et al., 2014). Taxa with different relative abundances have been shown to be driven by different environmental factors (Pedrós-Alió, 2012; Liu et al., 2015). Other studies have provided evidence that taxonomic resolution, which can detect evolutionary forces, may influence the strength of community-environment relationship (Lu et al., 2016). Here, we hypothesized that factors affecting the different bacterial communities are not the same and that the impacts of niche divergence or niche conservatism based on observed evolutionary patterns or scales are different.

In this study, high-throughput sequencing of 16S rRNA gene (V3–V5 regions) was used to investigate the soil bacterial community in the mangrove nature reserve of the Jiulong River Estuary in Fujian province, southeast China. We aimed to compare the influence of *S. alterniflora* invasion on bacterial community composition and function, and determine the key factor for driving microbial community assembly at different relative abundances, different niche breadths and different taxonomic resolutions. Particularly, we aim to answer the following key questions: how do belowground bacterial community composition, diversity, function change after *S. alterniflora* invasion in mangrove wetlands? Which taxa are most sensitive to *S. alterniflora* invasion? Are the controlling factors, and their contribution to the variation of community, different based on the analysis of relative abundance and taxonomic resolution?

## Materials and methods

### Study site and sampling

This study was carried out in a mangrove nature reserve on the Jiulong River Estuary (117°53′-117°55′E, 24°25′-24°29′N) in Fujian province, southeast China (Figure S1). In this subtropical coastal wetland, the dominant plant is the mangrove *Kandelia obovata*, however *Spartina alterniflora* has invaded a large area over the past few decades. All sampling stations and study design also featured in our previous studies on other aspects of this system-biogenic elements (Yu et al., 2015) and microeukaryotic community (Yu et al., 2014b). In the current study, all sampling stations were located in the intertidal zones where sediments are not always covered with water. Sediments samples were collected from four different types of habitats, i.e., unvegetated bare mudflat, cordgrass invaded zone with *S. alterniflora*, ecotone area with *S. alterniflora* and mangrove growing mixed together in the same area and native mangrove zone in four seasons, spring (April 2010), summer (August 2010), autumn (November 2010), and winter (January 2011). All samples were collected from the top 0–5 cm layer in sediment using a polyvinyl chloride (PVC) pipe (7 cm in diameter) and transported to the laboratory. In total, 16 sediment samples were collected from four stations across four seasons. We treated four different seasons as replicates, because bacterial communities among four seasons could not be significantly distinguished and our previous study confirmed that the microbial communities were relatively stable over four different seasons based on three replicates for each sample (Yu et al., 2014b). Each sample was freeze-dried at −55°C, then homogenized, filtered through a 150 μm mesh and finally stored at −80°C until further analysis.

### Measurement of environmental parameters

Salinity and pH of sediment porewater were measured by an ATAGO digital salt meter (Japan) and a Starter 2C pH meter (China), respectively. Total carbon (TC), total nitrogen (TN), total sulfur (TS), total phosphorus (TP), ammonium nitrogen (NH_4_-N), nitrite and nitrate nitrogen (NO_x_-N) concentrations were measured following standard methods used in our previous study (Yu et al., 2015). Detail information about environmental factors can be found in supplementary information (Figure S2).

### DNA extraction, PCR and 454 pyrosequencing

Total DNA was extracted and purified from 0.5 g of dry sediment using the FastDNA SPIN Kit and the FastPrep Instrument (MP Biomedicals, Santa Ana, CA, USA) following the manufacturer's instructions. All DNA samples were checked for quality using the agarose gel electrophoresis and quantified using the NanoDrop ND-100 device (Thermo Fisher Scientific, Waltham, MA, USA). The V3-V5 hypervariable region of 16S rRNA gene was amplified using the 357F (CCTACGGGAGGCAGCAG) and 926R (CCGTCAATTCMTTTRAGT) primer pair followed the protocol as described by Yu e al. (2014a). Each primer set used for PCR amplification contained an eight base DNA barcode for the multiplexing of samples in the pyrosequencing runs. In 50 μl reactions containing 1 μl of the primer set (10 μm each), 0.125 μl (5 U/μl) of Ex Taq DNA polymerase (Takara Bio, Otsu, Shiga, Japan), 2.5 μl of Ex Taq buffer (20 mM Mgcl_2_), 2 μl of deoxyribonucleotide triphosphate mixture (2.5 mM each, Takara Bio, Otsu, Shiga, Japan) and 50 ng of DNA template, PCR was performed included an initial denaturation at 94°C for 4 min, 25 cycles of 30 s at 94°C, 45 s at 50°C, 1 min at 72°C, and a final elongation step for 8 min at 72°C. Pyrosequencing was performed with Genome Sequencer FLX Titanium emPCR Kit (Lib-L) on a Roche 454 GS FLX+ Instrument according to the manufacturer's protocols (454 Life Sciences, Branford, CT, USA).

### Bioinformatics

Pyrosequencing flowgrams data were converted to sequence reads using the standard software provided by 454 Life Sciences. All the raw sequence data were processed in QIIME software packages (Caporaso et al., 2010). The sequences were quality-controlled using the split_libraries.py with following settings: 200 < sequence length < 1,000, mean quality > 25, ambiguous bases < 1, and homopolymer length < 6. Sequences were denoised using AmpliconNoise algorithm (shhh.seqs) in MOTHUR v.1.33.3 (Schloss et al., 2009; Quince et al., 2011; Liu et al., 2014). The sequences were then analyzed using pick_otus.py script (based on 97% sequence similarity). Chimeric sequences were checked by the ChimeraSlayer and then removed prior to further analysis (Schloss et al., 2009; Haas et al., 2011). Sequences were taxonomically classified using an 80% confidence threshold against the RDP Database (Yu et al., 2014a). All Archaea, Eukaryota, chloroplasts, mitochondria and unknown sequences and singleton OTUs were removed before the downstream analyses. Finally, in order to compare the community pattern between samples, the sequence data were normalized to 10,342 sequences per sample. The final cleaned sequence data set retained 165,472 reads and 9,749 OTUs at 97% sequence similarity level after trimming and quality filtering.

All sequence data, generated using Roche's 454 GS FLX+ system have been submitted to the Short Read Archive (SRA) database at National Center for Biotechnology Information (NCBI) (http://www.ncbi.nlm.nih.gov/) under the BioProject number PRJNA415893 and the accession number SRP122000.

### Definition of rare, conditionally rare, and abundant taxa

Microbial communities normally consist of a few abundant, and many rare species (Pedrós-Alió, 2012). Defining the rare biosphere is of somewhat arbitrary (Lynch and Neufeld, 2015). In this study, the thresholds for rare, conditionally rare, and abundant taxa were defined based on relative abundance cut-offs, with reference to recent publications (Liu et al., 2015; Chen et al., 2017; Dai et al., 2017). Rare taxa were defined as the OTUs with a relative abundance always < 0.01% in all samples. Conditionally rare taxa were defined as the OTUs being rare (relative abundance < 0.01%) in some samples but never being abundant (relative abundance ≥1%). Abundant taxa were defined as the OTUs that do not fall in either rare or conditionally rare categories. To reduce the effect of arbitrary definition of abundant and rare OTUs, multivariate cutoff level analysis (MultiCoLA) was used to evaluate how our data sets were influenced by different definitions (Gobet et al., 2010).

### Definition of habitat specialists, generalists and strict habitat specialists

The “Niche breadth” approach (Levins, 1968) was used to measure habitat specialization using the formula:

$$\begin{array}{l} B_{j}=\frac{1}{\overset{N}{\underset{i=1}{\sum }}P_{ij}^{2}} \end{array}$$

where *B*_*j*_ represents niche breadth and *P*_*ij*_ indicates the percentage of individuals belonging to species *j* present in a given habitat *i*. OTUs with mean relative abundances ≥2 × 10^−5^ were considered in this study, as these taxa probably indicate specialized taxa (Pandit et al., 2009). Habitat generalists will have a higher *B*-value and be more evenly distributed along a wider range of habitats compared with habitat specialists. In this study, there are four types of vegetation zones with each type representing a given habitat. OTUs with *B* > 3 were arbitrarily defined as generalists, but OTUs with *B* < 1.5 were defined as specialists. *B* > 3 was selected because this value lies within the outlier area of the *B* distribution. *B* < 1.5 was chosen as it is close to 1, the smallest possible *B*-value.

To identify strict habitat specialists for each type of vegetation, we performed indicator species analysis (ISA). Data used in the ISA were similar to those used in the analysis of “Niche breadth,” and samples were partitioned to mudflat, cordgrass, ecotone and mangrove as explained in the above section. Phylotypes with a *P*-value < 0.05 and both, a fidelity and specificity value ≥ 0.8, were considered as a good threshold for strict habitat specialists (Dufrene and Legendre, 1997).

### Analyses of community diversity

Rarefaction curves, ACE, Chao 1, Shannon-Wiener, Simpson and Pielou's evenness indices were calculated using MOTHUR v.1.33.3 (Schloss et al., 2009). One-way analysis of variance (ANOVA) was used to test the effects of vegetation and season on these indices by SPSS 20.0 (IBM Corp., Armonk, NY, USA).

Bray-Curtis similarity matrices within and between vegetation types were constructed using the bacterial community data based on relative abundance and database annotation, respectively. To compare the stability of habitats, coefficient of variation (CV) of Bray-Curtis dissimilarity across vegetation types or seasons was calculated. The non-metric multidimensional scaling (NMDS) ordination was performed using Bray-Curtis similarity matrices to investigate differences in bacterial community composition among samples using PRIMER 7.0 (Clarke and Gorley, 2015). Analysis of similarities (ANOSIM) was used to evaluate the significant differences between groups. No separation is indicated by *R* = 0, whereas *R* = 1 suggests complete separation (Clarke and Gorley, 2015). Spearman's rank correlations were used to determine the relationships between the Bray-Curtis similarity of bacterial community, Euclidean distance of environmental variables and the geographical distance of sampling sites, respectively.

### Relationships between community composition and environmental variables

Preliminary detrended correspondence analysis (DCA) of bacterial community data showed that the longest gradient lengths were shorter than 3.0, thus redundancy analysis (RDA) was used for further analysis. All environmental variables except pH were log(x+1) transformed to improve normality and homoscedasticity. Monte Carlo permutation tests were applied to evaluate the effect of environmental variables on variations in the soil bacterial community. Environmental factors with variance inflation factors (VIF > 20) were deleted to avoid collinearity. Variation partitioning analysis (VPA) was performed on the basis of RDA. We quantified the pure and shared influences of three groups of explanatory variables (environmental variables, season, and vegetation) on bacterial community composition variations. The residual fraction accounted for unexplained variation. DCA, RDA, and VPA were run in R using the vegan package (version 3.3.2) (R Development Core Team, 2015).

The distance-based redundancy analysis (Legendre and Anderson, 1999; Peres-Neto et al., 2006) was used to quantify the strength of community-environment relationships along taxonomic ranks by using the “capscale” function of “vegan” package in R (version 3.3.2) (R Development Core Team, 2015). Environmental factors used were similar to RDA analysis when we conducted distance-based redundancy analysis to equitably compare environmental influence on community composition along taxonomic ranks.

### Predicted functional profiles

To predict bacterial functional responses to the *S. alterniflora* invasion, we used PICRUSt (phylogenetic investigation of communities by reconstruction of unobserved states; http://picrust.github.com; Langille et al., 2013) to generate a functional profile using our 16S rRNA gene data. We followed the suggested methods for OTU picking with Greengenes v. 13.5 using Galaxy (http://huttenhower.sph.harvard.edu/galaxy/). KEGG (Kyoto Encyclopedia of Genes and Genomes) orthology group levels 2 and 3 of the predicted gene family abundances were compared using NMDS, ANOSIM and heat map, respectively.

### Neutral community model

To determine the potential importance of neutral processes to the whole bacterial community, we used Sloan's neutral community model (NCM) to predict the relationship between OTU detection frequency and their relative abundance along taxonomic ranks (Sloan et al., 2006). This neutral model can incorporate the influences of demographic and random dispersal processes (Logares et al., 2013). In this model, the parameter *R*^2^ determines the overall fit to the neutral model. The binomial proportion 95% confidence intervals around the model predictions were analyzed using the Wilson score interval in R using HMisc and minpack.lm packages (Logares et al., 2013).

## Results

### Alpha diversity of bacterial communities

A total of 165,472 high-quality sequences reads were obtained from all samples collected in the Jiulong River estuary, and were clustered into 9,749 operational taxonomic units (OTUs) based on 97% similarity level. The estimated species accumulation curves based on the pooled data set indicated that the majority of the bacterial taxa had been recovered from the studied sites, although none of single samples showed a full saturation in the rarefaction curve (Figure S3).

**Figure 1:**
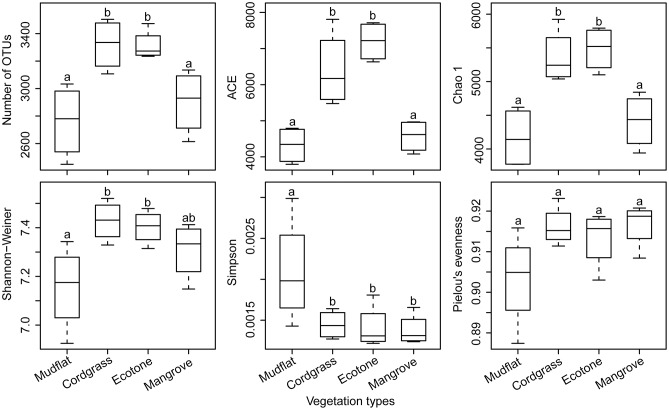
Comparison of alpha-diversity of the soil bacterial communities among four different types of vegetation zones. Ecotone indicates a transition zone with the distributions of cordgrass and mangrove overlap. The operational taxonomic units (OTUs) were defined at 97% sequence similarity threshold. Significant differences (*P* < 0.05) between vegetation types are indicated by different letters of the alphabet. Statistical analysis is Student's *t*-test with Bonferroni correction (*n* = 4). The ends of the box represent the 25th and 75th percentiles, the whiskers represent minimum and maximum range, and the center lines represent the median.

The one-way ANOVA indicated that vegetation types had significant effects on the number of OTUs, ACE, Chao 1, and Shannon-Wiener indices, but no significant difference of these alpha-diversity indices was found across four seasons (Table S1). Further, the community richness indices (number of OTUs, ACE, and Chao 1) in both partial and full cordgrass invasion zones were significantly higher than at the mudflat and mangrove stations, which indicates that cordgrass invasion may increase the alpha-diversity and richness of the soil bacterial community (Figure 1). However, Pielou's evenness showed no significant difference between the four types of vegetation zones or four seasons (Figure 1 and Table S1).

Conditionally rare taxa comprised of 7,516 OTUs (77.10%) and 127,233 sequences (76.89%) were the most diverse and dominant among three categories (abundant, conditionally rare, and rare), whereas 96 (0.98%) OTUs with 32,364 sequences (19.56%) were defined as being in the abundant taxa category, and 2,137 (21.92%) OTUs with 5,875 (3.55%) sequences were defined as rare taxa (Table S2). The multivariate cutoff level analysis showed that our definitions of abundant (19.56%) and rare (3.55%) bacteria are reasonable within the limitations of existing technology (Figure S4).

### Variations of bacterial taxonomic structure

**Figure 2:**
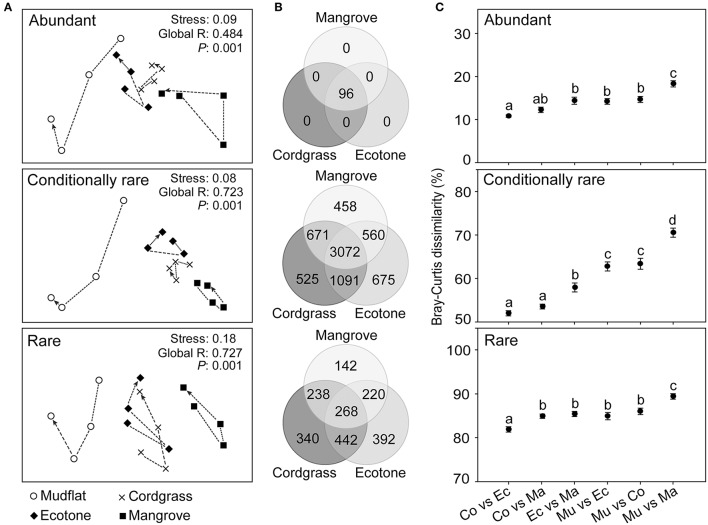
Comparison of beta-diversity of the soil bacterial communities among different types of vegetation zones. **(A)** Non-metric multidimensional scaling (NMDS) ordination of soil bacterial communities based on the Bray-Curtis dissimilarity. Points are connected by dash lines according to the progression of time (from spring to winter). **(B)** Venn diagram showing the number of OTUs (96 abundant OTUs, 7,052 conditionally rare OTUs, 2,042 rare OTUs) that are unique and shared between three different types of vegetation zones. **(C)** Pairwise Bray-Curtis dissimilarity of bacterial communities among different vegetation types. Mu, mudflat; Co, cordgrass; Ec, ecotone; Ma, mangrove. Significant differences (*P* < 0.05) between vegetation types are indicated by different letters of the alphabet. Statistical analysis is Student's *t*-test with Bonferroni correction (*n* = 16). Data are means ± standard error (error bars).

**Figure 3:**
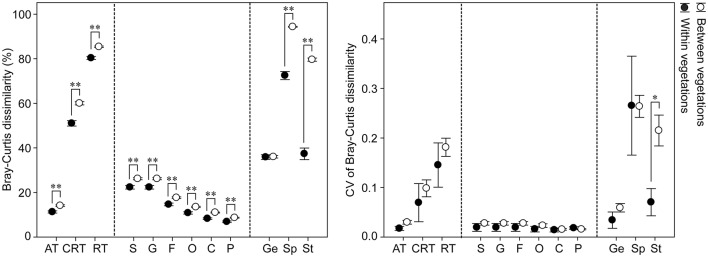
Pairwise Bray-Curtis dissimilarity of soil bacterial community and its coefficient of variation (CV) measured at different relative abundances, different taxonomic resolutions and different niche breadths. The bacterial community analyses are conducted on four vegetation zones: mudflat (*n* = 4), cordgrass (*n* = 4), ecotone (*n* = 4) and mangrove (*n* = 4). AT, abundant taxa; CRT, conditionally rare taxa; RT, rare taxa. S, species; G, genus; F, family; O, order; C, class; P, phylum. Ge, generalists; Sp, specialists; St, strict specialists (indicator species). Significance is calculated by nonparametric Mann-Whitney *U*-test. ^*^P < 0.05, ^**^P < 0.01. Data are expressed as means ± standard error (error bars).

**Figure 4:**
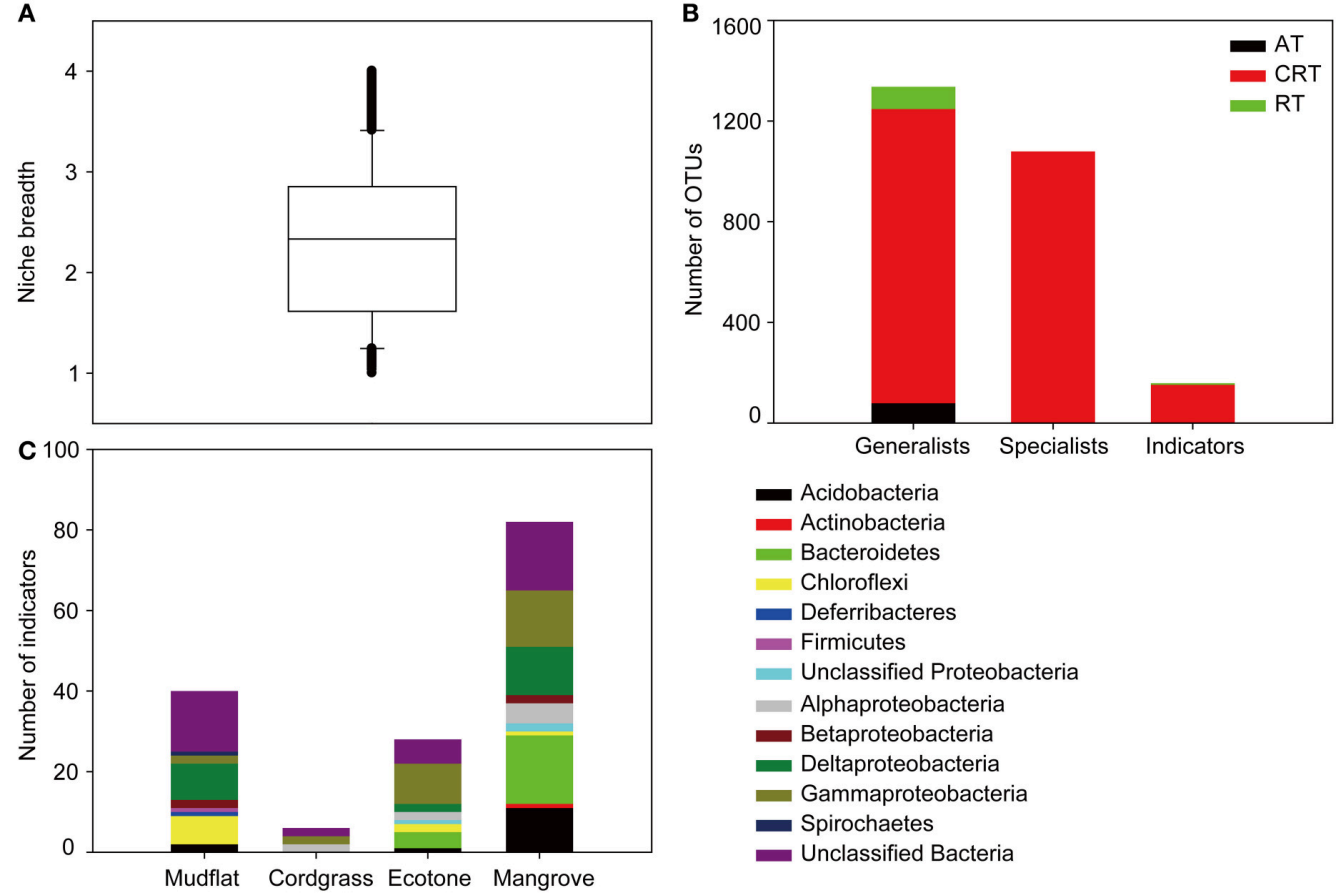
Habitat specialization of different OTUs based on niche breadth and INDVAL (INDicator VALues) analysis. **(A)** Distribution of niche breadth values of all selected OTUs. **(B)** The number of generalists, specialists, and strict habitat specialists (indicators) belonged to abundant, conditionally rare and rare taxa. OTUs with niche breadth value >3 were arbitrarily defined as generalists, whereas those with niche breadth <1.5 were selected as specialists. For the indicators, phylotypes with a *P*-value < 0.05 and both, a fidelity and specificity value ≥0.8, were considered as a good threshold for strict habitat specialists (Dufrene and Legendre, 1997). AT, abundant taxa; CRT, conditionally rare taxa; RT, rare taxa. Indicators, strict specialists. **(C)** The number and taxonomic composition of strong indicator taxa for the specific vegetation zones. Four habitats were mudflat, cordgrass, ecotone, and mangrove.

Our results revealed that the differences between bacterial communities could be attributed to vegetation type rather than seasonality or spatial effect (Figure 2). The entire bacterial community showed a significant negative correlation (*r* = −0.554, *P* < 0.01) with the geographical distance, while the environmental variables did not show any significant relationship (*r* = −0.074, *P* = 0.42) with the geographical distance (Figure S5). The divergences of bacterial community were very high and highly variable at different relative abundances (abundant, conditionally rare and rare) and niche breadths (generalists, specialists, strict specialists), however it was relatively low and showed a gradual decrease along taxonomic ranks from species to phylum (Figure 3). In terms of relative abundance, the conditionally rare subcommunity showed a striking separation compared with abundant and rare assemblies, confirmed by the pairwise Bray-Curtis dissimilarity of bacterial communities among different vegetation types (Figure 2C), and the analysis of similarity (ANOSIM) comparisons between soil bacterial subcommunities (Table 1). At niche breadths level, we identified 1,336 OTUs (13.7%) as habitat generalists and 1,079 OTUs (11.1%) as habitat specialists. Interestingly, habitat generalists were present among abundant, conditionally rare and rare taxa, whereas habitat specialists were only present in conditionally rare and rare taxa (Figure 4B). Based on INDVAL analysis, 158 OTUs (1.6%) were identified as strict habitat specialists (Figure 4). The numbers of these strict specialist OTUs among vegetation types were 40 in mudflat zone, 6 in cordgrass zone, 29 in ecotone zone, and 83 in native mangrove zone, respectively. The taxonomic compositions of strict specialists were significantly different among four types of vegetation zones (Figure 4C).

**Table 1:** Analysis of similarity (ANOSIM) results for comparisons between soil bacterial communities in different types of vegetation zones.

**Groups**	**Abundant taxa**	**Conditionally rare taxa**	**Rare taxa**
Mudflat vs. Cordgrass	0.573^*^	0.729^*^	0.667^*^
Mudflat vs. Ecotone	0.500^*^	0.677^*^	0.688^*^
Mudflat vs. Mangrove	0.875^*^	0.906^*^	0.969^*^
Cordgrass vs. Ecotone	0.500	0.583^*^	0.396
Cordgrass vs. Mangrove	0.385^*^	1.000^*^	0.844^*^
Ecotone vs. Mangrove	0.635^*^	0.990^*^	0.990^*^

*The operational taxonomic units (OTUs) were defined at 97% sequence similarity threshold*.

*Values show the R-value, and asterisks denote significant difference at the P < 0.05 level*.

*The ANOSIM statistic compares the mean of ranked dissimilarities between groups to the mean of ranked dissimilarities within groups. An R-value close to “1” suggests dissimilarity between groups while an R-value near “0” suggests an even distribution of high and low ranks within and between groups, respectively*.

Seven major phyla (Proteobacteria, Acidobacteria, Actinobacteria, Bacteroidetes, Cyanobacteria, Firmicutes, and Chloroflexi) were observed in this study. Proteobacteria (abundant 12.5 ± 0.5% vs. conditionally rare 35.7 ± 1.2% vs. rare 1.6 ± 0.1% based on the whole community) was the most dominant phylum in all subcommunities (Figure S6). At OTU level, 2,402 OTUs (24.6%) were shared among four different vegetation types, and most of the unique OTUs belonged to either conditionally rare taxa or rare taxa (Figure S7). All abundant taxa (96 OTUs) were shared among cordgrass, ecotone, and mangrove zones (Figure 2B).

The comparisons among the coefficient of variation (CV) of Bray-Curtis dissimilarity metrics showed that bacterial communities showed more stability between taxonomic ranks than at different relative abundances (abundant, conditionally rare and rare) or niche breadths (generalists, specialists, strict specialists) (Figure 3).

### Predicted functions of bacterial communities

The predicted functional distribution was grouped roughly based on the vegetation types (Figure S8), indicating the strong influence of cordgrass (*Spartina alterniflora*) invasion on bacterial functional groups. The predicted bacterial community function in mudflat or ecotone zone was significant different from mangrove zone (Table S3).

### The factors associated with variation of bacterial community

**Figure 5:**
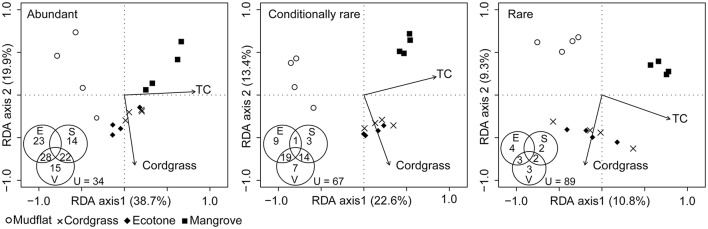
RDA ordination showing the bacterial community composition in relation to significant vegetation and soil properties (*P* < 0.05). All environmental factors were used in this analysis except those with variance inflation factors higher than 20 (VIF > 20). Cordgrass represents for *Spartina alterniflora*. TC, total carbon. Inside Venn diagram showed results of variation partitioning analysis, illustrating the effects of environment (E), vegetation (V) and season (S) factors on the community composition of soil bacteria. Values indicate the percentage of variation explained by each fraction, including pure, shared explained and unexplained (U) variability. Note that the fraction of variation values <1% are not shown for simplicity.

The RDA ordination showed that the abundant, conditionally rare and rare taxa subcommunities were significantly correlated with total carbon (TC) and cordgrass (*Spartina alterniflora*) according to forward selection model (*P* < 0.05; Figure 5). However, interpretation of the first two RDA dimensions for community variability substantially decreased from abundant (58.6%) to conditionally rare (36.0%) and rare (20.1%) taxa subcommunities. Our variation partitioning indicated that the impact of environmental, vegetational, and seasonal factors contributed to the structure of abundant, conditionally rare and rare sub-communities to different degrees (Figure 5). The vegetation was the most important factor, followed by environmental and seasonal factors for these three bacterial subcommunities. In addition, simultaneous effects of environmental and vegetation factors or seasonal and vegetation factors jointly accounted for the variation in bacterial subcommunities, as calculated by the sum of the shared fractions.

**Figure 6:**
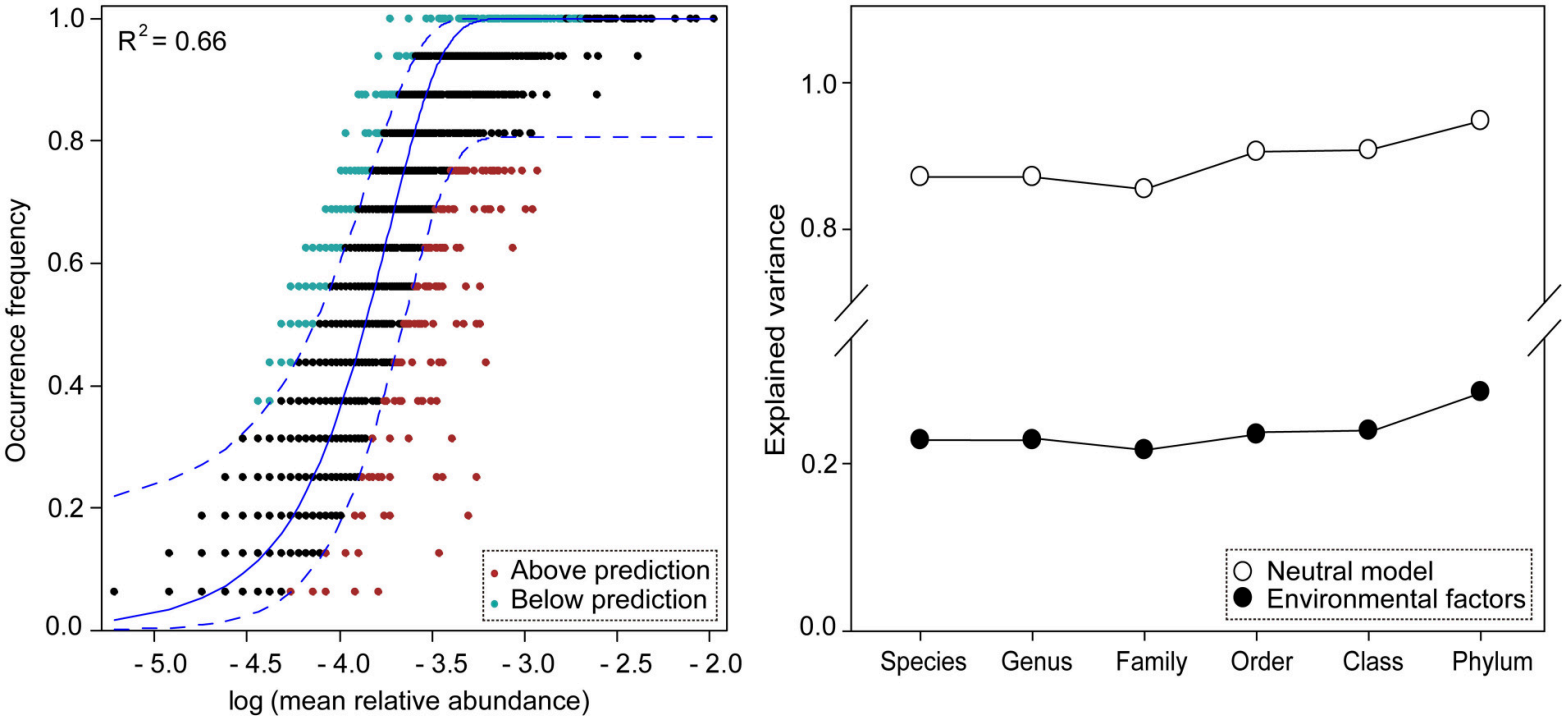
The variation in soil bacterial metacommunity explained by neutral model and environmental variables, respectively. **(Left)** Fit of the neutral model based on the entire bacterial communities from all vegetation zones (*n* = 9,749). Frequency of occurrence of different OTUs as a function of mean relative abundance based on 10,342 reads per samples in all data sets. Dash lines represent 95% confidence intervals around the model prediction (blue line). OTUs that occur more or less frequently than predicted by the model are shown in different colors. **(Right)** Comparison of explained community variations between neutral model and environmental factors along the taxonomic ranks. Taxonomy-based compositional variation is calculated based on our database annotation from species to phylum. The soil bacterial community analyses are conducted on 16 samples from Jiulong River estuary.

Interestingly, the neutral model successfully described the frequency of whole bacterial taxa in the different vegetation zones across four seasons (*R*^2^ = 0.66) (Figure 6). Further, the explained variation of neutral model and environmental selection tends to remain relatively constant along taxonomic ranks from fine to broad taxonomic levels, indicating that taxa within the same lineages generally show similar responses to environmental variations (Figure 6).

## Discussion

### Invasion effects on alpha-diversity of bacterial community

Our results clearly supported the view that cordgrass (*Spartina alterniflora*) invasion has positive effects on the alpha-diversity of soil bacterial community (Figure 1). So far, studies have suggested that the effects of cordgrass invasion on ecological diversity are complicated and inconsistent: having either negative, positive or no effect. For example, *S. alterniflora* invasion had negative effects on the alpha-diversity of macrobenthos (Wan et al., 2009), meiofauna (Lin et al., 2015), and nirS-containing denitrifiers (Zhang et al., 2013a), in contrast it had positive effects on the bacteria associated with the cordgrass roots (Hong et al., 2015) and nirK-containing denitrifiers (Zhang et al., 2013a). Several reasons could account for this positive effect on microbial diversity. First, the influence of *S. alterniflora* invasion on the diversity of the microbial community may depend on the community composition and structure. It has been shown that *S. alterniflora* invasion had negative, positive and insignificant effects on the diversity of methanogens, nirK-containing denitrifiers and sulfate-reducing bacteria, respectively (Zeleke et al., 2013; Zhang et al., 2013a; Yuan et al., 2016). Composition and structural variations within the entire bacterial community may offset each other. Second, different extents of invasion may lead to different results. It has been confirmed that severe plant invasions can increase mycorrhizal fungal abundance and diversity in a field experiment (Lekberg et al., 2013). This may imply that different invasion stages could result in distinct results. Also, the different sampling sites, such as endophytes vs. rhizoplane, or different latitudes, showed different results. Endophytes have been proven to be more sensitive to plant invasion than rhizosphere bacteria (Hong et al., 2015). In addition, rhizosphere bacterial diversity (Shannon-Weiner diversity index and number of DGGE bands) of *S. alterniflora* populations increased along a latitudinal gradient (Nie et al., 2010). It is important to note that the development of sequencing methods now allows a much more direct identification of the rare biosphere community in an environment (Pedrós-Alió, 2012). Conditionally rare and rare taxa, which are minor contributors to total community abundance, had important contributions to the higher diversity in both partial and full invasions of cordgrass (Figure S6 and Table S2). Moreover, the diversity of soil bacterial communities has been linked to functionally significant processes (Wolfe and Klironomos, 2005; Wagg et al., 2014; Jing et al., 2015). Ecosystem multifunctionality is positively associated with the diversity of soil bacterial community (Jing et al., 2015) thus the increased diversity in our study may indicate a functional increase or change in *S. alterniflora* invasion zone.

### Variations of bacterial community composition and functional predictions

Our results suggest that the bacterial communities were highly variable at different relative abundances (abundant, conditionally rare, and rare taxa) and niche breadths (generalists, specialists, strict specialists), however community variability was relatively low and stable among the taxonomic ranks (Figure 3). Consistent with our current knowledges of the effects of *S. alterniflora* (Nie et al., 2010; Hong et al., 2015), we found that bacterial community significantly changed after invasion. However, within this general result there were also some interesting differences in the influence on the bacterial community. Our results help integrate previous studies that have been based on different relative abundant taxa (Liu et al., 2015; Dai et al., 2017), niche breadths (Levins, 1968; Logares et al., 2013), and taxonomic ranks (Lu et al., 2016), in response to *S. alterniflora* invasion. For differentially abundant OTUs, our results supported the hypothesis that taxa with different relative abundances do not respond equally to the *S. alterniflora* invasion. Conditionally rare and rare taxa had a stronger separation between groups than abundant taxa indicating a stronger influence of *S. alterniflora* invasion on these taxa (Figure 2). This difference in response could be explained in two ways. On the one hand, abundant taxa with high density had stronger probabilities of dispersal compared with the conditionally rare and rare taxa, thereby resulting in a widespread or ubiquitous distribution (Liu et al., 2015; Figure 4B). On the other hand, rare taxa-which were mainly structured by local environmental variables were more susceptive to the environmental variations (Pedrós-Alió, 2012; Shade et al., 2014; Lynch and Neufeld, 2015). For niche breadth, many habitat generalists (1,336 OTUs) and specialists (1,079 OTUs) were found in soil bacterial community (Figure 4), similar to what has been reported by Logares et al. (2013) for bacterioplankton. A total of 158 strict habitat specialists were found closely associated with different vegetation types. Invasion may increase habitat diversification, and the habitats are likely to be filled by a series of habitat specialists which can change the community composition and function. In brief, long-term *S. alterniflora* invasion could generate stable new niches that were filled by a series of new habitat specialists but not suitable for mangrove specialists. The relatively steady community variations along taxonomic ranks may plausibly be an artifact of limited annotation information in the database.

Cordgrass invasion not only changed the structure of bacterial community but also altered community function. Previous studies have indicated that the community structure of related functional microorganisms was transformed after *S. alterniflora* invasion, and the carbon and nitrogen cycles were influenced in the estuary ecosystem (Hong et al., 2015). In this study, the predicted functional communities were separated roughly based on the vegetation types (Figure S8). When contrasting bacterial community and predicted functional variability in the soil, higher community variability with relatively stable functional distribution was found (Table 1 and Table S3). This is in accordance with a recent report on a marine system, where functional categories were found to be stably distributed across different zones, while community compositions varied significantly across zones (Sunagawa et al., 2015). Functional redundancy across different taxa in bacterial communities could explain this phenomenon and be regarded as a buffering capacity for an ecosystem resilience.

### Community assembly of soil bacteria

Several questions still remain unanswered in relation to the rare biosphere: particularly, which factors drive the variation in bacterial communities, and to what extent do these factors which influence the bacterial communities distributions vary with taxonomic resolutions (Logares et al., 2013; Liu et al., 2015; Lu et al., 2016).

It had already been shown that vegetation types, environmental factors, and seasons can drive microbial community structure in different types of ecosystems (Berg and Smalla, 2009; Zhang et al., 2013a; Yu et al., 2014b). Our variation partitioning analysis clearly showed the largest contribution to the variation in bacterial communities was from vegetation (Figure 5). This suggests that vegetation is the main force in shaping the soil bacterial community composition. Previous studies have shown that vegetation types can influence the bacterial community by several mechanisms-including complex effects such as the alteration of the properties of soil, litter quantity and quality, root exudates, transformation of the local microclimate or direct interactions with root-symbiotic microorganisms (Wolfe and Klironomos, 2005; Hui et al., 2017). In our study, *S. alterniflora* invasion had substantial effects on the properties of soil in the subtropical coastal wetland with the chemical characterization clustered by vegetation types (Figure 5; Yu et al., 2015). Some of the explanatory community variations were shared by both vegetation and environment. This may be due to the fact that vegetation can change the properties of the soil, so indirectly affect the bacterial community. In line with Sinha et al. (2009), we found that total carbon (TC) has a significant influence on the bacterial subcommunities (Figure 5). Vegetation can provide organic matter through leaf-litter inputs or through the release of root exudates into the soil environment (Wolfe and Klironomos, 2005).

However, the influence of environment (explanatory extent) on variations of bacterial subcommunities reduced from abundant, conditionally rare to rare taxa based on the partial RDA (Figure 5). One explanation for this may be the larger number of sequences of single specie for abundant taxa and their distinct ecological niches and different functions in ecosystem (Pedrós-Alió, 2012; Kim et al., 2013; Liu et al., 2015). Importantly, we found a constant strength for the community composition-environment relationships from fine to broad taxonomic levels (Figure 6), suggesting phylogenetic niche conservatism (the tendency of species to retain many of their ancestral ecological characteristics, Wiens and Graham, 2005). These findings further indicate that a broader taxonomic classification could strengthen niche-related signals, balance the distribution uncertainty associated with finer taxonomic units and support the recent view which suggest that same bacterial clades generally maintain similar ecological characteristics over evolutionary time (Martiny et al., 2015).

The unexplained community variation in variation partitioning analysis may be due to unmeasured abiotic variables (such as tide or irradiance) and biological variables (e.g., predator or virus). The tide can directly influence the bacterial species dispersal and could influence variations of physical and chemical parameters, which in turn influence microbial community dynamics (Yu et al., 2015). Moreover, based on neutral theory, ecological drift (stochastic processes of birth, death, colonization, and extinction) and evolutionary drift (stochastic genetic diversification) could also contribute to unexplained variation (Hanson et al., 2012; Zhou et al., 2014; Chen et al., 2017). Our results indeed showed that the neutral model successfully explained the 66% variations of bacterial community, indicating a strong role of stochastic processes (Figure 6). Altogether, community variation can also arise from an interaction mechanism between stochastic and deterministic processes due to the coexistence of multiple environmental gradients in the study areas.

## Conclusions

Our results demonstrated that invasion by *Spartina alterniflora* had significant effects on the soil bacterial community composition, diversity and function in an estuarine system. Our results suggested that bacterial communities were highly variable at different relative abundances (abundant, conditionally rare, and rare taxa) and niche breadths (generalists, specialists, strict specialists), however community variation was relatively low and stable among the taxonomic ranks. Conditionally rare and rare bacteria subcommunities exhibited a stronger response to cordgrass invasion than abundant subcommunity, although the higher proportion of community variance was explained by cordgrass and total carbon for abundant taxa. *S. alterniflora* invasion may promote habitat diversification, which is likely to lead to a loss in mangrove specialists and an increase in cordgrass specialists among the bacteria. All habitat specialists and strict specialists were either conditionally rare or rare bacteria. Both environmental selection and neutral process play very important roles in the community assembly, while vegetation is the main force in shaping the soil bacterial community composition. Due to the existence of a large number of rare microbial species in natural ecosystems, future studies based on deeper high-throughput sequencing, longer time-series sampling strategy, more complete information about soil physiochemical profile and function genes analyses will be needed to improve our understanding of the invasive species and effects on the wetland ecosystem.

## Author contributions

JY designed the research; ML, ZY, and XY performed the experiments; JY contributed the new reagents/analytic tools; ML and JY analyzed the data. All authors discussed the interpretation of the results and wrote the manuscript.

### Conflict of interest statement

The authors declare that the research was conducted in the absence of any commercial or financial relationships that could be construed as a potential conflict of interest.
