# Supplementary material for: Invasion by Cordgrass Increases Microbial Diversity and Alters Community Composition in a Mangrove Nature Reserve
Source: Front Microbiol. 2017 Dec 15;8:2503. doi: 10.3389/fmicb.2017.02503 (PMC5737034; doi:10.3389/fmicb.2017.02503)
Supplement: Supplementary file 1 [file DataSheet1.pdf]

Invasion by Cordgrass Increases Microbial Diversity and Alters Community Composition in a Mangrove Nature Reserve

Min Liu[1,2,3], Zheng Yu[1,4], Xiaoqing Yu[1], Yuanyuan Xue[1,2,3], Bangqin Huang[3] and Jun Yang[1*]

[1]*Aquatic EcoHealth Group, Key Laboratory of Urban Environment and Health, Institute of Urban Environment, Chinese Academy of Sciences, Xiamen 361021, China,* [2]*University of Chinese Academy of Sciences, Beijing 100049, China,* [3]*College of the Environment and Ecology, Xiamen University, Xiamen 361102, China,* [4]*Department of Chemical Engineering, University of Washington, Seattle 98105, USA*

[*]For correspondence: Jun Yang, Aquatic Ecohealth Group, Key Laboratory of Urban Environment and Health, Institute of Urban Environment, Chinese Academy of Sciences, Xiamen 361021, People's Republic of China. E-mail: jyang@iue.ac.cn; Tel. (+86)5926190775; Fax (+86)5926190775.

Supplementary Material

Summary

The supplementary material includes 8 figures and 3 tables.

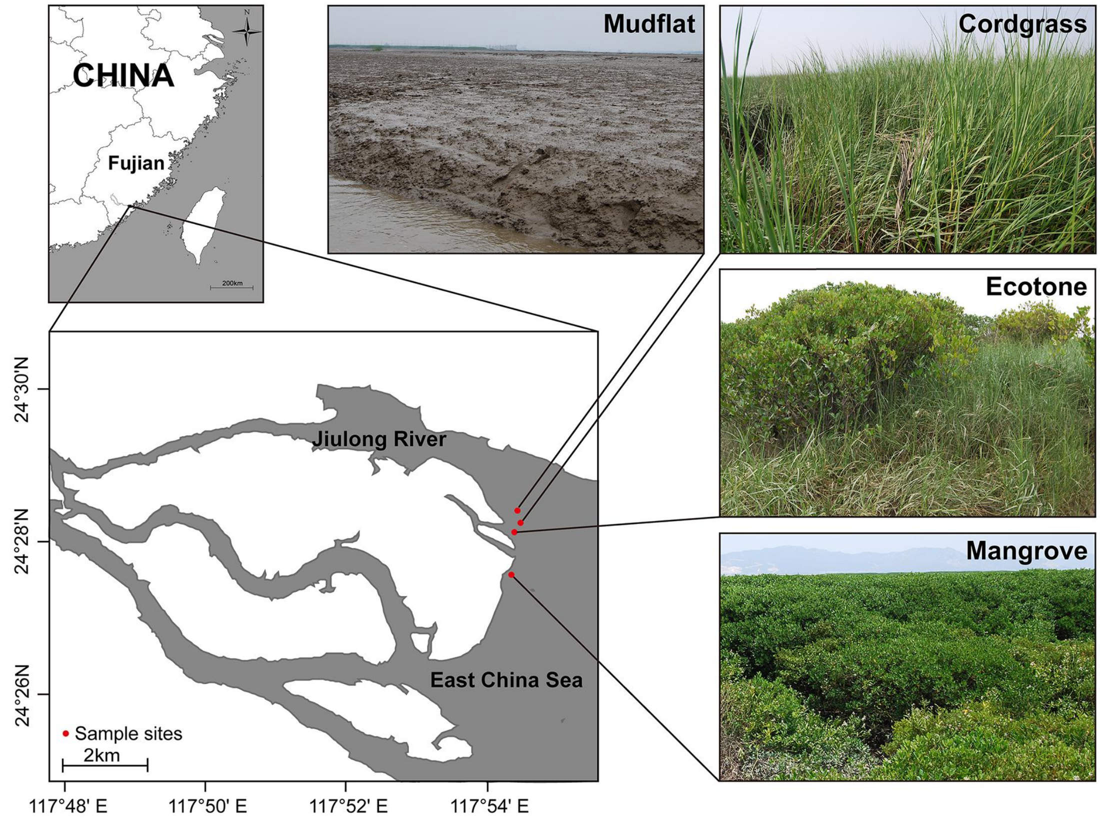

FIGURE S1 | Map of Jiulong River estuary showing the sampling locations and their vegetation types.

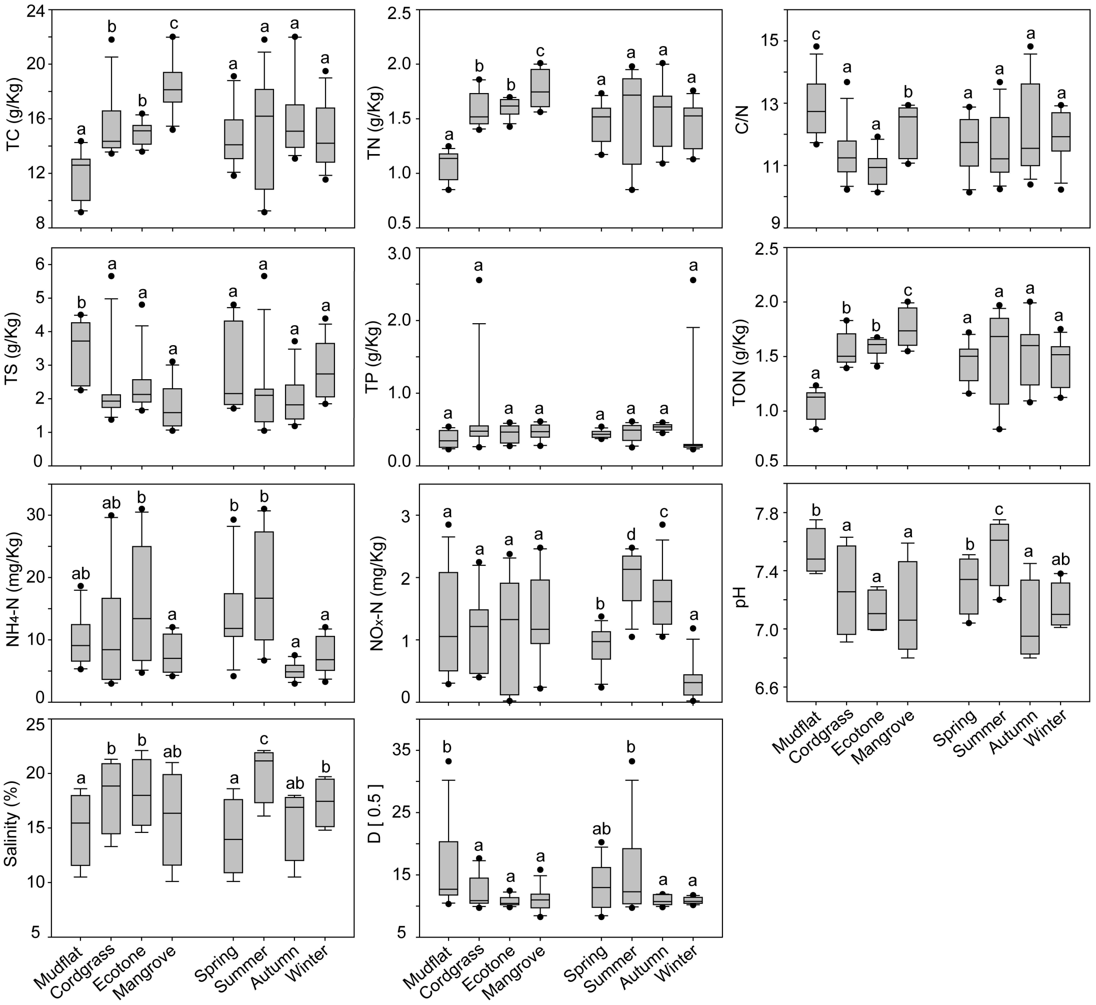

FIGURE S2 | Boxplots showing the spatiotemporal changes in environmental variables in different vegetation zones or different seasons. Significant differences (*P* < 0.05) are indicated by different letters of the alphabet. Statistical analysis was Student's t test with Bonferroni correction (n = 4). The ends of the box represent the 25th and 75th percentiles, the whiskers represent minimum and maximum range, black dots represent outliers and the center lines represent the median.

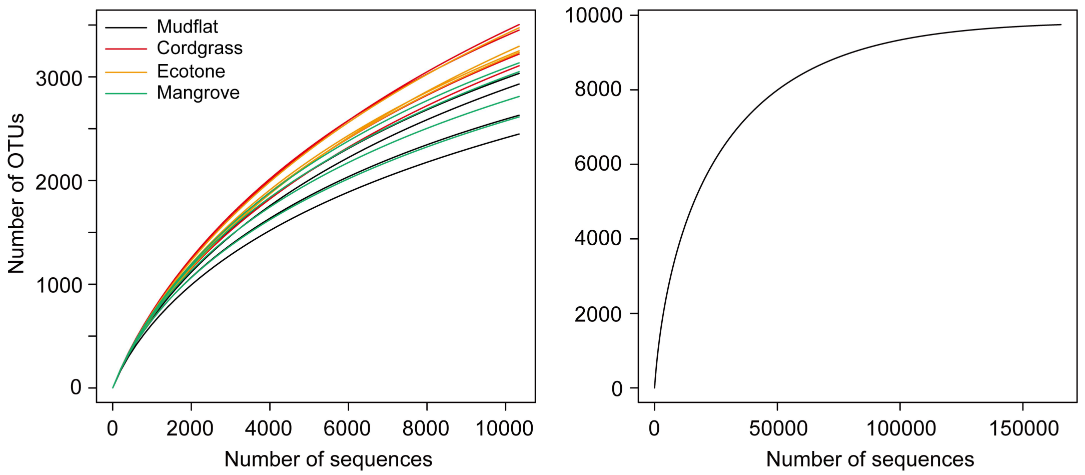

FIGURE S3 | Rarefaction curves of similarity-based operational taxonomic units (OTUs) at 97% sequence similarity level. Left - the individual samples, right - the combined set of 16 samples.

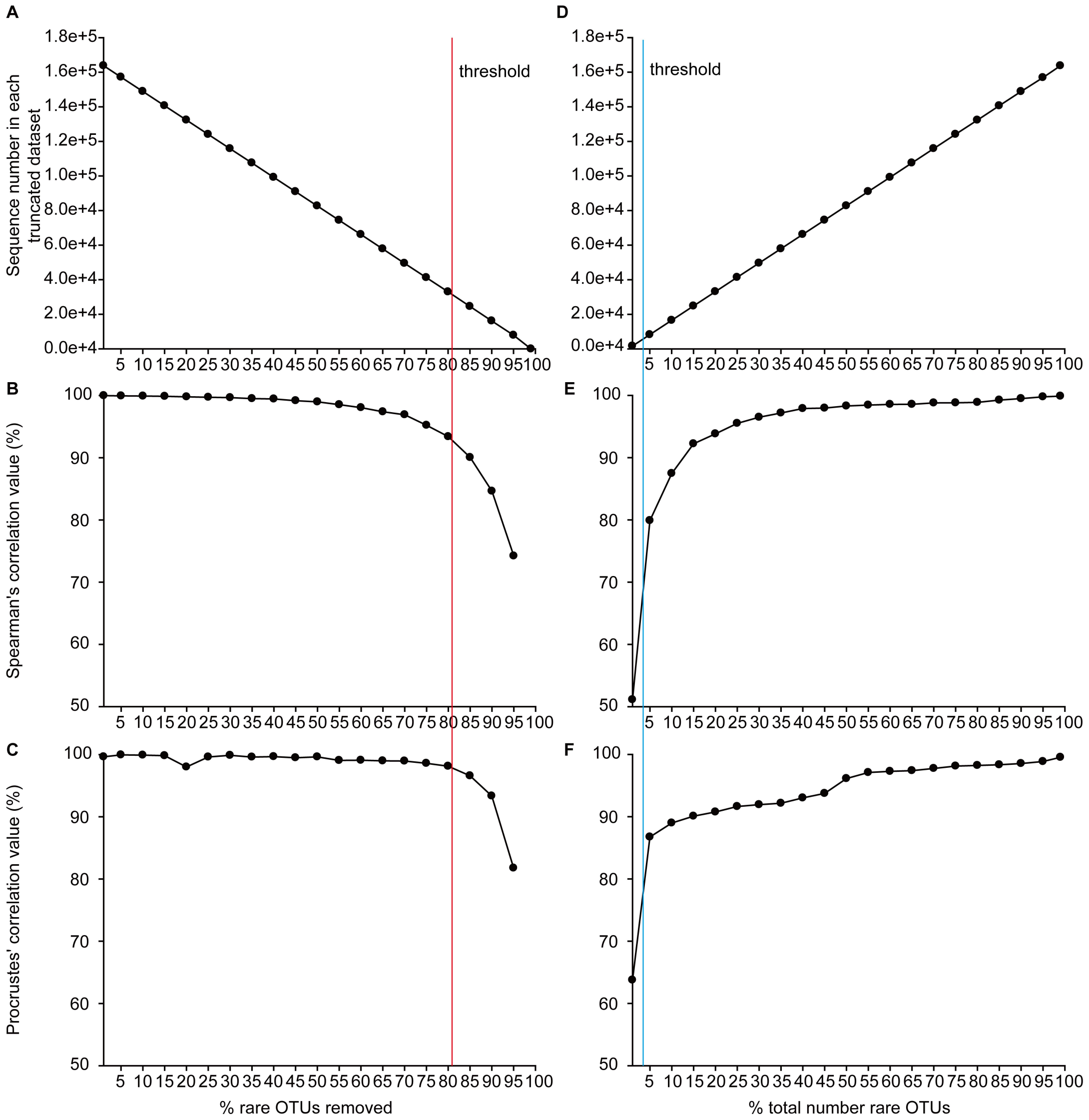

FIGURE S4 | MultiCoLA profiles based on the dataset-cutoff approaches. The rare OTUs were removed (**A–C**), and the rare OTUs were retained (**D–F**) in each truncated dataset. Abundance of OTUs in each truncated dataset **(A, D)**. Non-parametric Spearman correlations comparing the deviation in complete data structure between the original matrix and truncated matrices **(B, E)**. Comparison of most important axes of extracted variation between the original and truncated datasets **(C, F)**. Missing points are due to removal of samples from applying a given cutoff to the original dataset. The red line indicated the threshold of abundant OTUs (19.56%), and the blue line indicated the threshold of rare OTUs (3.55%) in this study, respectively.

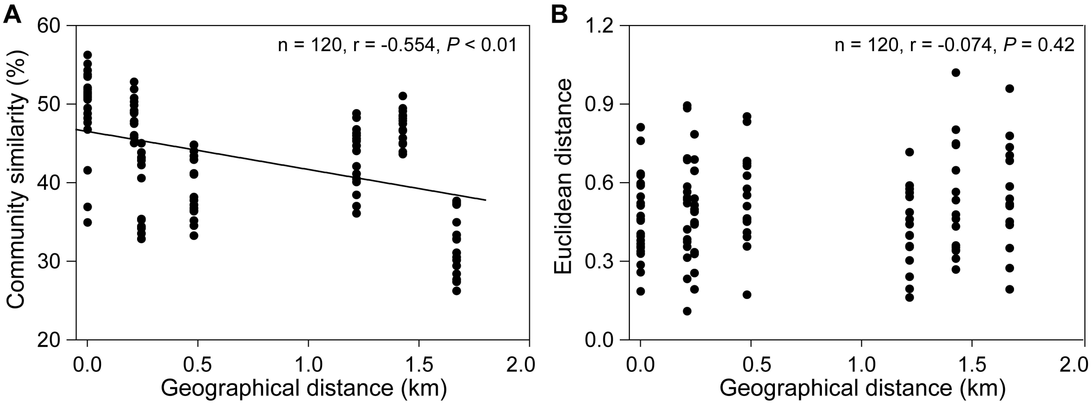

FIGURE S5 | Spearman's rank correlations between the Bray-Curtis similarity of the bacterial community and geographical distance (**A**), Euclidean distance of all environmental variables and geographical distance (**B**), respectively (n is the number of comparisons, all environmental variables were used, see materials and methods).

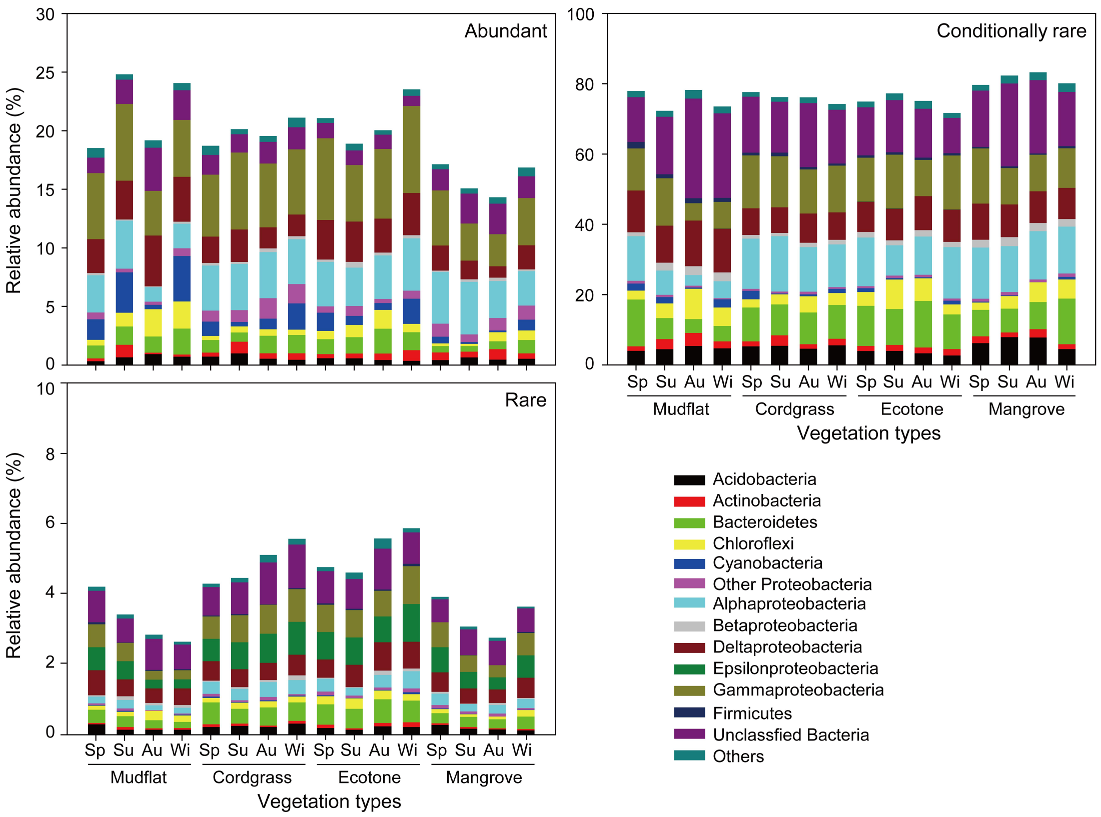

FIGURE S6 | Relative abundance of major bacterial taxa across four seasons in four different types of vegetation zones. The low relative abundance phyla were merged as "others". Sp, spring; Su, summer; Au, autumn; Wi, winter.

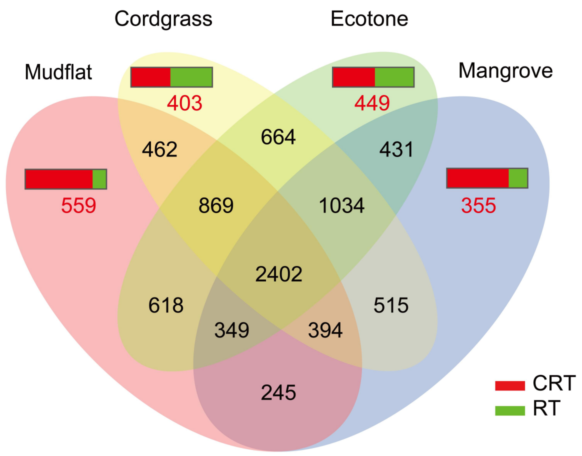

FIGURE S7 | Venn diagram showing the number of OTUs that are unique and shared among four different types of vegetation zones. Numbers in red represent the unique OTUs in specific vegetation zone. Histograms represent the ratio that the unique OTUs belong to abundant (AT), conditionally rare (CRT) and rare taxa (RT). Note that no any abundant OTU was restricted to one specific vegetation zone.

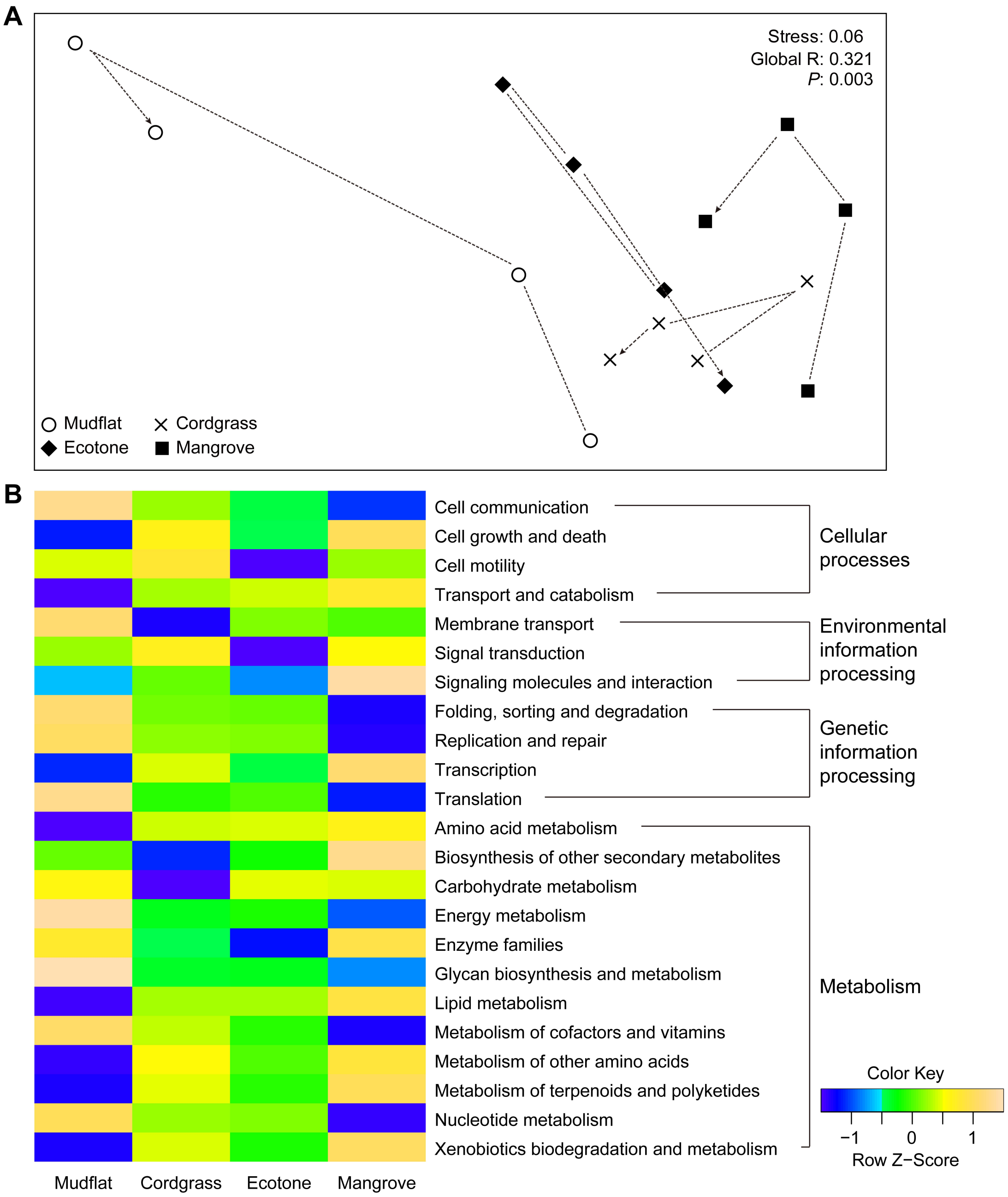

FIGURE S8 | Results of Phylogenetic Investigation of Communities by Reconstruction of Unobserved States (PICRUSt). **(A)** Non-metric multidimensional scaling (NMDS) analysis based on the calculated Bray-Curtis dissimilarities between the functional gene family abundances of 16 samples. Points are connected by dash lines according to the time (from spring to winter). **(B)** Heatmap of differentially average abundant KEGG pathways showing shifts in bacterial functional profiles among different vegetation types. KEGG pathways with obvious increasing or decreasing trends are displayed in yellow or blue colors, respectively. To simplify analysis, however, only tier 1 functions of "metabolism," "genetic information processing," "environmental information processing" and "cellular processes" were analyzed further, as the categories of "organismal systems" and "human disease" were thought to be poorly relevant to environmental samples.

TABLE S1 | Results of one-way ANOVA testing the effects of vegetation and season on bacterial alpha-diversity.

**Factors**	**Vegetation**			**Season**		
	**df**	**F**	***P***	**df**	**F**	***P***
Number of OTUs	3	7.458	0.004**	3	0.155	0.924
ACE	3	16.449	0.000**	3	0.153	0.926
Chao 1	3	10.841	0.001**	3	0.203	0.893
Shannon-Wiener	3	4.482	0.025*	3	0.399	0.756
Simpson	3	3.197	0.062	3	1.082	0.394
Pielou's evenness	3	1.474	0.271	3	2.118	0.151

The operational taxonomic units (OTUs) were defined at 97% sequence similarity threshold.

* $P < 0.05$; ** $P < 0.01$.

TABLE S2 | General description of abundant, conditionally rare, rare and total taxa data sets at 97% similarity level.

Category	Description	OTU number	Sequence number
Abundant taxa (AT)	Always abundant taxa	0	0
	Conditionally abundant taxa	9 (0.09%)	8510 (5.14%)
	Conditionally rare and abundant taxa	4 (0.04%)	1716 (1.04%)
	Moderate taxa	83 (0.85%)	22138 (13.38%)
		96 (0.98%)	32364 (19.56%)
CRT	Conditionally rare taxa	7516 (77.10%)	127233 (76.89%)
RT	Rare taxa	2137 (21.92%)	5875 (3.55%)
Total	All taxa	9749 (100%)	165472 (100%)

Always abundant taxa were defined as the OTUs with a relative abundance always ≥ 1% in all samples.

Conditionally abundant taxa were defined as the OTUs with a relative abundance greater than 0.01% in all samples and ≥ 1% in some samples but never rare (< 0.01%).

Conditionally rare and abundant taxa were defined as the OTUs with a relative abundance varying from rare (< 0.01%) to abundant (≥ 1%).

Moderate taxa were defined as the OTUs with relative abundance between 0.01% and 1% in all samples.

Conditionally rare taxa (CRT) were defined as the OTUs with a relative abundance < 0.01% in some samples but never ≥ 1% in all samples.

Rare taxa (RT) were defined as the OTUs with a relative abundance always < 0.01% in all samples.

In this study, the abundant taxa included always abundant taxa, conditionally abundant taxa, conditionally rare and abundant taxa, and moderate taxa based on the results of multivariate cutoff level analysis (For details see Supplementary Figure S4).

TABLE S3 | Analysis of similarity (ANOSIM) results for comparisons between differentially abundant KEGG pathways identified of bacterial functional profiles in different types of vegetation zones.

**Groups**	**Global R**	***P***
Mudflat vs Cordgrass	0.354	0.086
Mudflat vs Ecotone	0.281	0.114
Mudflat vs Mangrove	0.625*	0.029
Cordgrass vs Ecotone	0.271	0.114
Cordgrass vs Mangrove	0.281	0.114
Ecotone vs Mangrove	0.385*	0.029

The operational taxonomic units (OTUs) were defined at 97% sequence similarity threshold.

* $P < 0.05$.
